# Treatment of periscapular tendinopathy with radiofrequency coblation: A case report

**DOI:** 10.1177/2050313X20930612

**Published:** 2020-07-09

**Authors:** Alexander Tham, Michael McLean, Caroline Atherton, Neal L Millar

**Affiliations:** Institute of Infection, Immunity and Inflammation, College of Medical, Veterinary and Life Sciences, University of Glasgow, Glasgow, UK

**Keywords:** Tendinopathy, tendon, therapy, coblation, inflammation

## Abstract

Overuse injuries of the tendon – ‘tendinopathy’ – account for 30%–50% of all sporting injuries and a high proportion of orthopaedic referrals from primary care physicians. Tendinopathies often have a multifactorial aetiology and injury can be due to a combination of both acute and chronic trauma which contributes to loss of tissue integrity and eventual rupture. Our incomplete understanding of the mechanisms surrounding tendon pathophysiology continues to cause difficulties in treatments beyond loading regimes which can be unsuccessful in up to 30% of cases. We describe an uncommon case of tendinopathy affecting the periscapular muscle/tendon unit in a 35-year-old female with persistent pain around the inferior posterior pole of her right scapula. Magnetic resonance imaging findings confirmed oedema of the muscles around the inferior scapular margin in keeping with enthesopathy/tendinopathy and she was treated with radiofrequency coblation to the area. This case highlights radiofrequency ablation as a surgical option should non-operative treatments fail in the rare diagnosis of periscapular tendinopathy.

## Introduction

Overuse injuries of the tendon – encompassed by the term ‘tendinopathy’ – represent an underestimated group of musculoskeletal disorders accounting for annual cost of US$3 billion to the US healthcare system highlighting the huge burden of disease.^1^ The molecular mechanism contributing to the development of tendinopathies remains largely unknown; however, inflammatory pathways have recently been implicated in several functionally relevant model systems.^2^ Tendinopathy in the periscapular muscles is highly uncommon and is difficult to treat. Manifestations range from mild pain and swelling to complete loss of function and diagnosis is usually based on a thorough history and physical examination;^3^ however, imaging modalities such as ultrasound and magnetic resonance imaging (MRI) can be useful, especially for identifying tears.^4^ The main goal in tendinopathy treatment is to reduce pain and allow return to activity – first-line treatment comprising several modalities ranging from relative rest and progressive loading to invasive pharmacological interventions continues to be the mainstay of treatment,^3^ while surgical options remain the last option owing to their morbidity and inconsistent outcomes.^5^

Radiofrequency (RF)-based plasma treatment is where the charged plasma breaks down molecular bonds in soft tissues^6^ and has been previously used in musculoskeletal surgical procedures. When applied to tendons, it is called microtenotomy and it involves the ablation of small segments of the tendon and leaving the remainder intact; this stimulates release of angiogenic factors – vascular endothelial growth factor (VEGF) and α_V_ integrin – which promote tendon healing.^7^

## Case report

A 35-year-old Caucasian female presented with a 1-year history of persistent atraumatic onset of pain over the inferior corner of her right scapula. She was referred to our upper limb tendon clinic after persistent right scapular pain and tenderness of periscapular muscles at the point of insertion following previous non-resolution with physiotherapy instigated by her primary care physician.

Past medical history included left ulnar neuritis and anterior right knee pain. Prior procedures include right and left ovarian cystectomy and emergency appendectomy.

On examination, there was no evidence of muscle atrophy. She was able to forward elevate her right shoulder to 160° and 180° on contralateral left side; abduct to 160°, externally rotate to around 50° and internally rotate to T12 on affected side and T6 on contralateral side. There were no signs of impingement with unremarkable functioning tests on rotator cuff muscles and no sign of scapular winging. Point tenderness was over inferior aspect of medial and lateral borders of scapula.

**Figure 1.**
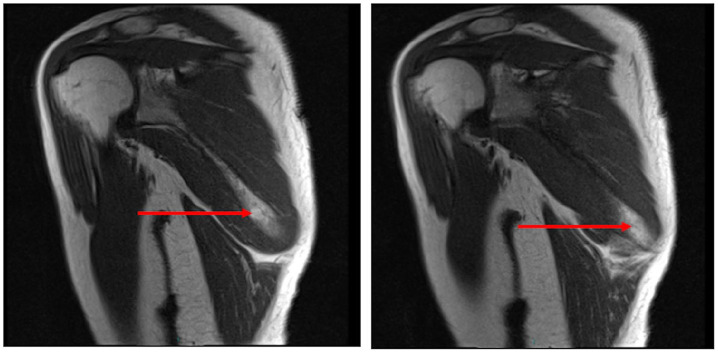
T1-weighted MRI images of right scapula. Standard sequences without contrast sagittal T1-weighted images of right scapula. A focal bone marrow oedema of approximately 2 cm is highlighted at the inferior margin and the inferior pole of the right scapula (red arrows). In addition, there is slight oedema of the adjacent muscles around the inferior scapular margin in keeping with enthesopathy/tendinopathy.

**Figure 2.**
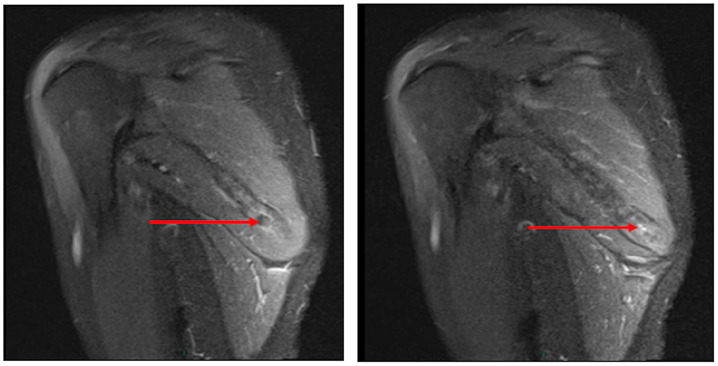
T2-weighted MRI images of right scapula. Standard sequences without contrast sagittal T2-weighted images of right scapula. Images confirm soft tissue oedema within the serratus anterior with residual bone marrow oedema within the inferior angle of the scapula in keeping with tendinopathy/enthesopathy (red arrows).

MRI scans of her right scapular region indicated ‘resolving enthesopathy’ with the presence of reduced bony oedema around inferior border of her scapular serratus anterior and teres major insertion (Figures 1 and 2). No muscle tear or distended scapulothoracic bursae were demonstrated on the MRI scan, and periscapular bulk was maintained.

Initial management involved the use of glyceryl trinitrate patches (GTN5 μg/24 h) along with referral to a shoulder physiotherapist for rotator cuff strengthening and periscapular muscle patterning exercises. The patient reported reduced symptoms with GTN patches at 8 weeks post commencing therapy; however, benefits subsided after stopping use for 6 weeks. Another 6-week course of GTN patches was continued, but the patient noted that the response had diminished.

Following these two injections into the periscapular region at the point of maximal tenderness with steroid (40 mg depomedrone) and local anaesthetic showed reduced symptoms for approximately 4 months. Intermittent numbness in right C5/C6 region was discovered with normal neurological tests and unremarkable findings on MRI of cervical spine. The patient was additionally referred to a rheumatology clinic.

**Figure 3.**
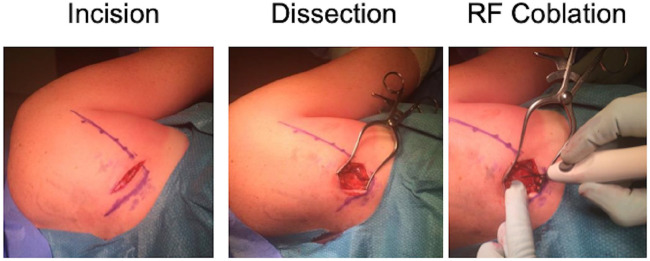
Surgical procedure of microtenotomy. Images illustrating initial incision over the posterolateral border of the scapula over the MRI highlighted area in Figure 1 and 2. Patient was positioned in the lateral decubitus position and the skin prepared with alcoholic chlorhexidine. The posterior interior lateral border of the scapula was marked as depicted. A small transverse incision (5 cm) was made with superficial dissection through fat and down to border of inferior pole of right scapula. The tendinous area of latissimus dorsi/teres major identified. Evidence of neovascularization, oedema and degenerative changes at insertion. Microtenotomy was carried out in a 2 cm × 2 cm grid area with 15 individual treatments.

Following discussion of the risk and benefits of surgery, the patient agreed to undergo RF ablation of the tendinous insertion of serratus anterior/teres major region. The surgery was carried out in the lateral decubitus position, and the skin was prepared with alcoholic chlorhexidine. No prophylactic antibiotics were administered. Posterior inferior lateral border of scapula was marked, and a small transverse incision was made (Figure 3). Dissection was carried out to border of scapula; tendinous area was identified and noted to have areas of neovascularization and degeneration. RF coblation was done in a grid (2 cm × 2 cm) of 15 small treatments. No adverse events were reported, and minimal blood loss was noted with the procedure taking approximately 30 min. The wound was closed with a 2/0 vicryl and 3/0 vicryl rapide. No active bleeders were identified prior to closure and drains were not used.

The patient was reviewed in clinic 2 weeks later and noted to have a fully healed wound without any surrounding erythema or discharge. Minor swelling was present at this point, and a reported 90% reduction in pain symptoms around right scapular region was noted by the patient. The patient was referred to ESP shoulder physiotherapy. At 10 weeks’ follow-up, a reported 80% improvement in symptoms with excellent range of motion and no tenderness were observed, and at 6 months, the patient was discharged from care with no return of symptoms and continued improvement of approximately 80%.

## Discussion

RF has been used historically to resect, coagulate or ablate tissue,^7^ and recently, coblation has been used for chondroplasty, meniscal repair, lateral epicondylitis, rotator cuff tendinopathy and tendinopathy of the foot and ankle.^8^ The plasma created is an electrically conducting gas made up of free electrons, ions and neutral chemical radicals and has sufficient energy due to the electrical fields to break down water molecules into excited H and OH radicals.^9^ By exciting water molecules, a chemical process begins which breaks molecular bonds in soft tissue at relatively low temperatures.^6^

When applied to tendons, it is termed microtenotomy and it involves the ablation of small segments of the tendon, leaving the remainder intact; this stimulates release of angiogenic factors – VEGF and α_V_ integrin – which promote tendon healing.^7^ Ahmed et al.^10^ postulated that tendon rupture occurs in the most hypovascular areas where the nutrition required by tenocytes for repair is compromised and thus cannot synthesize extracellular matrix to repair and remodel the damaged tendon.

In a study review by Tasto et al.,^7^ supraspinatus tendons from eight patients with rotator cuff tears were compared with six healthy tendons – the altered exhibited lack of vascularity, expressed by lower level of markers such as VEGF and α_V_ integrins. Another study performed RF-based microdebridement on the Achilles tendon of New Zealand white rabbits, demonstrating that coblation can trigger a healing response, providing increased tissue vascularity and number of organized fibroblastic cells.^11^ In another study of 20 patients, coblation was used for treatment in tendinopathy of Achilles tendon, patellar tendon and lateral epicondylitis – this showed improved pain symptoms in the short term.^8^

A clinical study examining a prospective case series showed that the RF-based plasma microtenotomy approach is safe and effective through at least 2 years for alleviating symptoms associated with chronic tendinosis in the lateral elbow.^8^ Another study comparing RF-based plasma microtenotomy with subacromial decompression in rotator cuff tendinosis noted that pain and functional symptoms associated with the condition were successfully treated through 1 year. Clinical outcomes in the RF group were equivalent to those receiving the ‘gold standard’ arthroscopic subacromial decompression.^12^

## Conclusion

Tendinopathy in the periscapular region of the shoulder is an uncommon location representing a challenging treatment dilemma. In this case report, we demonstrate that RF coblation is a viable treatment option for periscapular tendinopathy with good clinical outcomes. This is in line with clinical studies conducted, where RF-based microtenotomy has been used safely and effectively in Achilles tendinopathy, lateral epicondylitis and rotator cuff tendinopathy. We maintain that RF treatment may be a useful surgical adjunct in patients who have failed non-operative treatment for persistent periscapular tendon pathology.

## References

[1] Scott A Ashe MC Common tendinopathies in the upper and lower extremities. Curr Sports Med Rep 2006; 5(5): 233–241. 16934204 10.1097/01.csmr.0000306421.85919.9c

[2] Millar NL Murrell GAC McInnes IB Inflammatory mechanisms in tendinopathy – towards translation. Nat Rev Rheumatol 2017; 13(2): 110–122. 28119539 10.1038/nrrheum.2016.213

[3] Andres BM Murrell GAC Treatment of tendinopathy: what works, what does not, and what is on the horizon. Clin Orthop Relat Res 2008; 466(7): 1539–1554. 18446422 10.1007/s11999-008-0260-1 PMC2505250

[4] Peters JA Zwerver J Diercks RL, et al Preventive interventions for tendinopathy: a systematic review. J Sci Med Sport 2016; 19(3): 205–211. 25981200 10.1016/j.jsams.2015.03.008

[5] Bahr R Fossan B LÃ¸ken S, et al Surgical treatment compared with eccentric training for patellar tendinopathy (Jumper’s Knee). J Bone Joint Surg Am 2006; 88(8): 1689–1698. 16882889 10.2106/JBJS.E.01181

[6] Yeap EJ Chong KW Rikhraj IS Radiofrequency coblation for chronic foot and ankle tendinosis. J Orthop Surg 2008; 17(3): 325–330. 10.1177/230949900901700317 20065374

[7] Tasto JP Cummings J Medlock V, et al The tendon treatment center: new horizons in the treatment of tendinosis. Arthroscopy 2003; 19(Suppl 1): 213–223. 14673441 10.1016/j.arthro.2003.10.004

[8] Tasto JP Cummings J Medlock V, et al Microtenotomy using a radiofrequency probe to treat lateral epicondylitis. Arthroscopy 2005; 21(7): 851–860. 16012499 10.1016/j.arthro.2005.03.019

[9] Stadler S Gempel K Bieger I, et al Detection of neonatal argininosuccinate lyase deficiency by serum tandem mass spectrometry. J Inherit Metab Dis 2001; 24(3): 370–378. 11486903 10.1023/a:1010560704092

[10] Ahmed IM Lagopoulos M McConnell P, et al Blood supply of the Achilles tendon. J Orthop Res 1998; 16(5): 591–596. 9820283 10.1002/jor.1100160511

[11] Medlock VB Amiel D Harwood F, et al Cellular structural and angiogenic response to bipolar radiofrequency treatment of normal rabbit Achilles tendon. In: 49th Transactions of the Annual Meeting of the Orthopaedic Research Society, 2003, Poster #0819, https://www.ors.org/Transactions/49/0819.pdf

[12] Taverna E Battistella F Sansone V, et al Radiofrequency-based plasma microtenotomy compared with arthroscopic subacromial decompression yields equivalent outcomes for rotator cuff tendinosis. Arthroscopy 2007; 23(10): 1042–1051. 17916468 10.1016/j.arthro.2007.04.018

